# Variation in the stearoyl-CoA desaturase gene (*SCD*) and its influence on milk fatty acid composition in late-lactation dairy cattle grazed on pasture

**DOI:** 10.5194/aab-63-355-2020

**Published:** 2020-11-03

**Authors:** Yunhai Li, Huitong Zhou, Long Cheng, Jenny Zhao, Jonathan Hickford

**Affiliations:** 1 Department of Agricultural Sciences, Faculty of Agriculture and Life Sciences, Lincoln University, Lincoln 7647, New Zealand; 2 Faculty of Veterinary and Agricultural Sciences, Dookie College, The University of Melbourne, Victoria 3647, Australia

## Abstract

Gene markers have become useful tools for improving animal genetics and breeding since they improve the accuracy of selection for superior breeding stock. In this study, the stearoyl-CoA desaturase (Δ-9-desaturase) gene (*SCD*) was investigated in New Zealand pasture-grazed Holstein–Friesian × Jersey cows. Three nucleotide substitutions were identified in exon 5 of the gene (c.702A/G, c.762T/C and c.878C/T), and a single nucleotide substitution was identified in intron 5 (c.880+105A/G). The c.878C/T substitution would, if expressed, result in the amino acid substitution p.A293V. Four nucleotide substitutions (c.*1783A/G, c.*1883C/T, c.*1984G/A and c.*2066T/C/G) were identified in the 3${}^{\prime }$-untranslated region (3${}^{\prime }$-UTR), and these resulted in three nucleotide sequence variants (named a, b and c). The sequence that would encode valine (V) at position 293 of *SCD* was linked to 3${}^{\prime }$-UTR variant a, and the sequence that would encode alanine (A) was linked to variants b and c. The frequency of the genotypes was as follows: *VV* (equivalent to *aa*: 15.1 %), *VA* (equivalent to ab+ac: 50.0 %) and *AA* (equivalent to bb+cc+bc: 34.9 %). The cows with the V variant produced less C10:1, C12:1 and C14:1 fatty acid (FA) but more C10:0, C11:0, C14:0, C16:1 and C18:2 FA than the A variant cows (P<0.001). Effects of c.*1783A/G and c.*2066T/C/G on milk fat composition were also found for the *AA* cows. The presence of c was associated with decreased levels of C16:1 (P<0.001), C17:1 (P=0.001), C18:2 *cis*-9, *trans*-13 (P=0.045), C18:2 *cis*-9, *trans*-12 (P=0.018) FA and C16:1 FA index (P<0.001). The presence of b was associated with increased levels of C13:0 *iso* FAs (P<0.001), monounsaturated FA (MUFA; P=0.002) and C12:1 (P<0.001).

## 1 Introduction

Stearoyl-CoA desaturase (SCD), also named Δ9-desaturase, can introduce a double bond at the Δ9,10 position in a large spectrum of fatty acids (FAs), and it is a rate-limiting enzyme in catalysing the synthesis of monounsaturated fatty acids (MUFAs) from saturated FAs (SFAs) (Nakamura et al., 2004; Paton et al., 2009). The main substrates for SCD are C16:0 and C18:0 FA, which can be converted into C16:1 *cis*-9 and C18:1 *cis*-9 (Paton et al., 2009), but it can also catalyse the formation of *cis*-9, *trans-*11 conjugated linoleic acid (CLA) from C18:1 *trans*-11 (Ntambi et al., 2004).

The gene (*SCD*) encoding for SCD is located on bovine chromosome 26, and it is expressed in a variety of tissues (Chung et al., 2000). In lactating ruminants, the expression of *SCD* occurs at high levels (Bernard et al., 2005; Bionaz et al., 2008; McDonald et al., 1973), and it is considered to be important with respect to milk fat composition in these animals (Gautier et al., 2006). Ntambi et al. (2004) reported that dietary factors could regulate the expression of *SCD*, and hence the expression patterns of *SCD* in cows that are grazing outdoor on pasture might therefore be different to cows that are fed indoors on supplements. This is supported by the observation of Elgersma (2015) that milk from grazing-based production systems has less SFA and more polyunsaturated FA (PUFA), which is considered beneficial for health.

Other than the effect of dietary factors, nucleotide sequence variation in *SCD* is reported to be another factor that can change milk traits. For example, a nonsynonymous nucleotide substitution in *SCD* exon 5 (c.878C/T), which causes the substitution of valine (V) with alanine (A) at position 293 of the protein (p.A293V), has been associated with some milk traits.

While Carvajal et al. (2016) and Valenti et al. (2019) did not find associations between p.A293V and the gross milk traits of milk yield, fat percentage and protein percentage, associations were reported between p.A293V and the levels of individual FAs (C10:0 to C18:0) in Italian Holstein, Piedmontese and Valdostana cattle (Mele et al., 2007; Moioli et al., 2007). A higher frequency of the alanine allele of p.A293V was found in the Holstein cows (0.57) (Mele et al., 2007), Valdostana cows (0.65) (Moioli et al., 2007) and Jersey cows (0.94) (Moioli et al., 2007), while Carvajal et al. (2016) reported frequencies of 0.65, 0.81, 0.89, 0.56 and 0.92, in Holstein, Jersey, Frisón Negro, Montbéliarde and Overo Colorado cattle respectively. In these breeds, the *SCD* alanine allele was typically associated with a higher monounsaturated FA content.

In Japanese beef cattle, Taniguchi et al. (2004) identified more variants in the 3${}^{\prime }$-untranslated region (3${}^{\prime }$-UTR) of *SCD*, including c.*829C/T, c.*2066T/C/G, c.*2273G/A, c.*2458G/A and c.*3649A/T (these were labelled as 1905, 3143, 3351, 3537 and 4736 in their study), but the effect of these variations on intra-muscular fat composition was not significant, and their effect on milk FA is unknown. Hence, the objective of this study was to investigate the relationships between *SCD* variation in two regions – the first spanning p.A293V and the second spanning the nucleotide variation identified by Taniguchi et al. (2004) – and milk traits in Kiwicross™ cows during late lactation and in a wholly pasture-based outdoor dairy production system.

## 2 Materials and methods

### 2.1 Cows studied and milk sample collection

This research was approved by the Lincoln University Animal Ethics Committee (AEC Number 521) under the provisions of the Animal Welfare Act 1999 (NZ Government).

In total, 450 Holstein–Friesian × Jersey (HF × J) crossbred (Kiwicross™) dairy cows from two herds (124 cows in herd 1 and 326 cows in herd 2) were studied. The cows were between 3 to 10 years of age, and they were grazed solely outdoors on pasture (a mixture of perennial ryegrass and white clover) on the Lincoln University Dairy Farm (LUDF; Canterbury, NZ). The cows calved over the period August–September, and they were then milked twice a day until the end of May in the following year.

The milk yield in litres per day was recorded using Tru-test milk meters (Tru-test Ltd, Auckland, NZ). Milk samples for analysis were collected once a month from September to February, and these were analysed for fat percentage (%) and protein percentage (%) using Fourier-transform infrared spectroscopy (MilkoScan FT 120, Foss, Hillerød, Denmark). The milk samples for individual FA analysis were collected at one afternoon milking in mid-January (days in milk (DIM)=148±19 d) and frozen at -20 ${}^{\circ }C$. After freezing, the samples were freeze-dried and then individually ground to a fine powder for component analysis.

### 2.2 Gas chromatography of the fatty acids in the milk sample

Fatty acids in the milk samples were analysed by gas chromatography as FA methyl esters (FAME), as described in Li et al. (2019).

### 2.3 PCR-SSCP analysis and genotyping

A blood sample from each of the cows was collected onto FTA cards. These were air-dried and stored for analysis. For the genetic analysis, leukocyte DNA was purified from a 1.2 mm punch of the dried blood spot using the method described by Zhou et al. (2006).

Two pairs of PCR primers were designed based on the cattle reference sequence (NM_173959.4) to amplify two target regions of *SCD*. The first pair (forward: 5${}^{\prime }$-AATCAGGTAGGTCTCAGCG-3${}^{\prime }$ and reverse: 5${}^{\prime }$-TTCTAATACTGTCCCTTAG-3${}^{\prime }$) amplified a fragment of 436 bp (Region 1) spanning part of intron 4, exon 5 and part of intron 5. The second pair (forward: 5${}^{\prime }$-GAACCACTGTTTCTCTTTAC-3${}^{\prime }$ and reverse: 5${}^{\prime }$-CACTTTGGAACCTGCCTTTG-3${}^{\prime }$) amplified a fragment of 397 bp (Region 2) containing part of the 3${}^{\prime }$-UTR. The primers were synthesized by Integrated DNA Technologies (Coralville, IA, USA).

The PCR amplifications were performed as 15 µL reactions containing the purified genomic DNA on a 1.2 mm punch of the purified FTA paper, 0.25 µM of each designed primer, 150 µM of each dNTP (Bioline, London, UK), 2.5 mM of Mg${}^{2+}$, 0.5 U of Taq DNA polymerase (Qiagen, Hilden, Germany) and 1× the reaction buffer supplied with the DNA polymerase enzyme.

The amplifications were undertaken using S1000 thermal cyclers (Bio-Red, Hercules, CA, USA). The thermal profile included an initial denaturation for 2 min at 94 ${}^{\circ }C$, which was followed by 35 cycles of 30 s at 94 ${}^{\circ }C$, 30 s at 50 ${}^{\circ }C$ (for Region 1) or 56 ${}^{\circ }C$ (for Region 2) and 30 s at 72 ${}^{\circ }C$. A final extension was undertaken for 5 min at 72 ${}^{\circ }C$.

Following the PCR amplifications, a 0.7 µL aliquot of the products was mixed with 7 µL of loading dye (98 % formamide, 10 mM EDTA, 0.025 % bromophenol blue, 0.025 % xylene cyanol). After denaturation at 95 ${}^{\circ }C$ for 5 min and rapid cooling on wet ice, the samples were loaded on 16cm×18cm, 12 % acrylamide : bisacrylamide (37.5:1) (Bio-Rad) gels with 2 % glycerol. Electrophoresis was performed using Protean II xi cells (Bio-Rad), at 390 V for 19 h at 12 ${}^{\circ }C$ in 0.5× TBE buffer. The method of Byun et al. (2009) was used to stain the gels.

### 2.4 Sequencing of the *SCD* variants and sequence analysis

Homozygous PCR amplicons identified using PCR-SSCP were sequenced at the Lincoln University DNA Sequencing Facility. If homozygous samples were not found upon PCR-SSCP analysis, individual variants were isolated from heterozygous cattle and sequenced using an approach described previously (Gong et al., 2011). Briefly, single bands of interest from the heterozygous cattle were recovered directly from the SSCP gels as a gel slice. This was macerated and the DNA was eluted into 50 µL TE buffer by incubating at 70 ${}^{\circ }C$ for 20 min. The original primers and 1 µL of the eluted solution (as a template) were used for a second round of PCR amplification to produce a simple SSCP gel pattern which could be directly compared to or found in, the pattern derived from the original heterozygous cow. When banding patterns could be matched and identified, then the second homozygous PCR amplicons were directly sequenced at the Lincoln University DNA Sequencing Facility.

The computer program DNAMAN (version 5.2.10, Lynnon BioSoft, Canada) was used for sequence alignment and comparisons. The BLAST algorithm was used to search the NCBI GenBank database (https://blast.ncbi.nlm.nih.gov, last access: 23 January 2020) for homologous sequences.

### 2.5 Statistical analyses

Hardy–Weinberg equilibrium (HWE) for the *SCD* genotypes was analysed using an online chi-square calculator (http://www.husdyr.kvl.dk/htm/kc/popgen/genetik/applets/kitest.htm, last access: 2 April 2020). Other statistical analyses were carried out using IBM SPSS version 22 (IBM, NY, USA). Associations between variation in *SCD* and variation in gross milk traits and milk FA traits were tested using general linear mixed-effects models (GLMMs).

Single-variant presence/absence models (fixed effects: DIM, age and herd) were used to ascertain which variants should be analysed in subsequent multi-variant models. The multi-variant models included any variant that had a variant – gross milk trait or variant – FA trait association in the single-variant presence/absence analysis with a P value of less than 0.200 (and thus were potentially affecting the trait). The multi-variant models were again corrected for the fixed effects of (DIM, age and herd) and with other variants fitted as random effects.

A GLMM (fixed effect: genotype, DIM, age and herd) and multiple pair-wise comparisons with Bonferroni corrections were used to ascertain the effect of the different genotypes with a frequency greater than 5 % (thus insuring adequate sample size), on gross milk production traits (milk yield, fat percentage and protein percentage). Next, a GLMM (fixed effect: genotype, DIM, age and herd) and multiple pair-wise comparisons with Bonferroni corrections were used to ascertain the effect of genotypes on milk FA component levels.

The effect of cow sire could not be included in the GLMMs. Some semen straws (sire genetics) used in NZ dairy cattle artificial -insemination-based breeding approaches contain mixed-sire semen purchased from commercial semen producers. In these cases, individual sire identity is impossible to ascertain, but because the straws were mixed-semen straws and because different sires are used for different inseminations in different years, it is unlikely that sire was a strongly confounding effect. Cow age and herd might also be confounded with sire, but this cannot be confirmed.

## 3 Results

### 3.1 Variation in *SCD*

**Figure 1:**
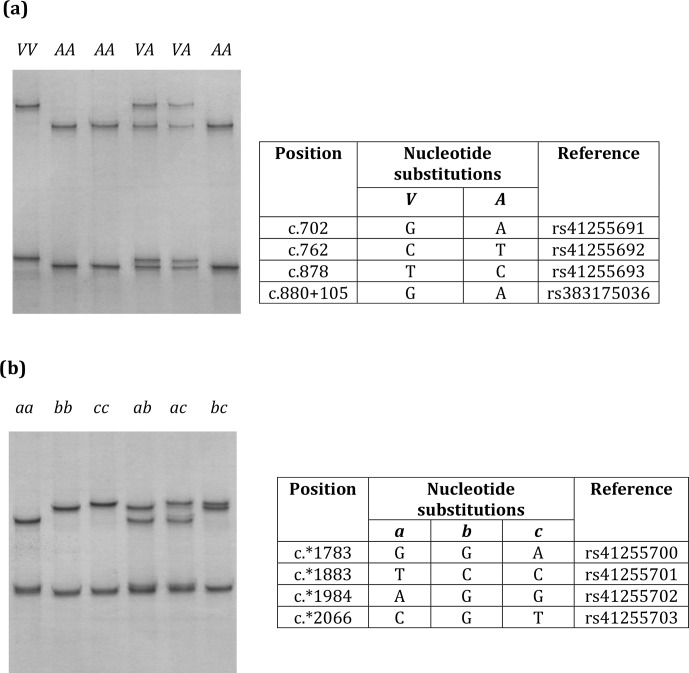
Variation in bovine *SCD*. Unique PCR-SSCP patterns representing different sequence variants of Region 1 **(a)** and Region 2 **(b)** are shown.

Eight nucleotide substitutions were found in the two regions of *SCD* investigated (Fig. 1).

Three nucleotide substitutions (c.702A/G, c.762T/C and c.878G/A) in exon 5 and one nucleotide substitution (c.880+105A/G) in intron 5 (Region 1) and four nucleotide substitutions (c.*1783A/G, c.*1883C/T, c.*1984G/A, and c.*2066T/C/G) in the 3${}^{\prime }$-UTR (Region 2) were found in the Kiwicross™ cows studied.

The variants A and V (from p.A293V), described in previous studies, were identified by these nucleotide variations (Fig. 1a), and three variants (a, b and c) could be identified when the 3${}^{\prime }$-UTR variations were analysed (Fig. 1b). A linkage was observed between c.702A/G, c.762T/C, c.878C/T, c.880+105A/G, c.*1883C/T and c.*1984G/A. The V variant in Region 1 corresponded to 3${}^{\prime }$-UTR variant a in Region 2, and the A variant in Region 1 corresponded to variants b and c in Region 2.

The overall frequency of the genotypes was *VV* (equivalent to *aa*: 15.1 %), *VA* (equivalent to ab+ac: 50.0 %) and *AA* (equivalent to bb+cc+bc: 34.9 %) across both herds, with the individual herds having frequencies of A (58.13 %) and V (41.87 %) for Herd 1 and A (60.35 %) and V (39.66 %) for Herd 2. Overall, the dominant variant was A (59.9 %) and the frequency of V was 40.1 %. The P value for the chi-square for deviation from HWE for p.A293V was 0.388, suggesting that across both herds the population was at equilibrium.

Six genotypes *aa*, *ab*, *ac*, *bb*, *bc* and *cc* were identified in the 3${}^{\prime }$-UTR region amplified, with overall frequencies of 15.1 %, 10.7 %, 39.3 %, 1.8 %, 10.7 % and 22.4 % respectively. The frequencies for the individual SNPs in both herds are illustrated in Table 1. The most common variant was c (47.4 %), and the frequencies of a and b were 40.1 % and 12.4 %. The P value for the chi-square for deviation from HWE was 0.724, suggesting that across both herds the population was at equilibrium.

**Table 1:** Frequencies of *SCD* 3${}^{\prime }$-untranslated region SNPs in the two herds of cows investigated.

SNP		Variant	Frequency (%)	
Position	Sequence		Herd 1	Herd 2
c.*1783	A	c	48.0	46.9
	G	a,b	52.0	53.1
c.*1883	C	b,c	58.1	60.4
	T	a	41.9	39.6
c.*1984	G	b,c	58.1	60.4
	A	a	41.9	39.6
c.*2066	T	c	48.0	46.9
	C	a	41.9	39.6
	G	b	10.1	13.5

### 3.2 Milk traits, milk fat compositions and *SCD* variation

The average phenotypic measures for the gross milk traits of milk yield, milk fat percentage and milk protein percentage were 20.86±0.389 $L\;d^{-1}$, 5.07±0.044 % and 4.22±0.024 % respectively for Herd 1. In Herd 2 they were 22.58±0.211 $L\;d^{-1}$, 5.06±0.031 % and 4.11±0.017 % respectively, but no associations were revealed between the *SCD* variation and these gross milk traits. In contrast, Table 2 summarizes the associations revealed between the composition of milk fat and the *SCD* variants defined by p.A293V.

**Table 2:** Association between milk fatty acid levels and p.A293V genotypes.

FAME	Mean FAME level ± SE${}^{1}$ ($g\;100\;g^{-1}$ milk FA)			$P^{2}$
	*VV* (n=68)	*VA* (n=225)	*AA* (n=149)	
C10:0	3.36±0.061${}^{a}$	3.23±0.049${}^{b}$	3.16±0.054${}^{b}$	**0.002**
C10:1	0.26±0.006${}^{c}$	0.29±0.005${}^{b}$	0.32±0.005${}^{a}$	<0.001
C11:0	0.06±0.003${}^{a}$	0.06±0.002${}^{b}$	0.06±0.003${}^{b}$	**0.011**
C13:0 *iso*	0.07±0.001${}^{c}$	0.08±0.002${}^{b}$	0.09±0.002${}^{a}$	<0.001
C12:1	0.09±0.003${}^{c}$	0.09±0.002${}^{b}$	0.10±0.003${}^{a}$	<0.001
C13:0	0.13±0.004${}^{a}$	0.12±0.003${}^{b}$	0.12±0.004${}^{b}$	**0.005**
C14:0	12.92±0.141${}^{a}$	12.53±0.112${}^{b}$	12.36±0.124${}^{b}$	<0.001
C14:1 *cis*-9	0.80±0.027${}^{c}$	0.98±0.021${}^{b}$	1.17±0.024${}^{a}$	<0.001
C16:1	1.45±0.039${}^{a}$	1.35±0.031${}^{b}$	1.25±0.034${}^{c}$	<0.001
C17:0 *iso*	0.54±0.011${}^{b}$	0.56±0.008${}^{a}$	0.58±0.009${}^{a}$	**0.001**
C17:1	0.21±0.004${}^{a}$	0.20±0.003${}^{\text{ab}}$	0.20±0.004${}^{b}$	**0.013**
C18:2 *cis-*9, *trans*-13	0.30±0.006${}^{a}$	0.29±0.005${}^{\text{ab}}$	0.28±0.005${}^{b}$	**0.004**
C18:2 *cis-*9,* trans-*12	0.08±0.004${}^{a}$	0.08±0.003${}^{a}$	0.07±0.003${}^{b}$	<0.001
MCFA	21.53±0.273${}^{a}$	20.88±0.218${}^{b}$	20.58±0.240${}^{b}$	**0.001**
C10:1 index	7.19±0.197${}^{c}$	8.30±0.157${}^{b}$	9.37±0.173${}^{a}$	<0.001
C12:1 index	2.09±0.051${}^{c}$	2.31±0.041${}^{b}$	2.53±0.045${}^{a}$	<0.001
C14:1 index	5.82±0.194${}^{c}$	7.27±0.155${}^{b}$	8.64±0.170${}^{a}$	<0.001
C16:1 index	3.72±0.086${}^{a}$	3.44±0.069${}^{b}$	3.18±0.076${}^{c}$	<0.001

${}^{1}$ Predicted means and standard error of those means derived from GLMM. “Cow age”, “days in milk” and “herd” were fitted to the models as fixed effects. Means within a row that do not share a superscript letter are separated by Bonferroni test at P<0.05. ${}^{2}$ P<0.05 in bold. MCFA: medium-chain fatty acid.

Tables 3 and 4 summarize the associations revealed between the *SCD* variants a, b and c and the composition of individual and grouped FAs respectively. The results are not presented if no association was found with the variants. Overall, the presence of variant a was associated with lower C10:1 index, C12:1 index, and C14:1 index values but elevated C16:1 levels. Variant b or c appeared to have an opposite effect on the milk FAs when compared to variant a. There were higher C10:1 index, C12:1 index, C14:1 index and C18:1 index values when variant b was present.

**Table 3:** Association between individual fatty acid levels and *SCD* 3${}^{\prime }$-UTR variation.

FAME	Variant	Other variants in	Mean FAME level ± SE${}^{1}$				$P^{2}$
		the model	($g\;100\;g^{-1}$ milk FA)				
			Absent	n	Present	n	
C10:0	a	None	3.16±0.053	157	3.26±0.047	293	**0.004**
	b	None	3.26±0.048	346	3.17±0.055	104	**0.035**
	c	None	3.28±0.053	124	3.21±0.049	326	*0.076*
	a	b,c	3.16±0.068	157	3.25±0.063	293	**0.030**
	b	a,c	3.26±0.069	346	3.16±0.073	104	**0.029**
	c	a,b	3.26±0.079	124	3.18±0.075	326	*0.067*
C10:1	a	None	0.32±0.006	157	0.28±0.005	293	<0.001
	b	None	0.28±0.005	346	0.31±0.006	104	<0.001
	c	None	0.28±0.006	124	0.30±0.006	326	<0.001
	a	b,c	0.32±0.018	157	0.29±0.018	293	<0.001
	b	a,c	0.29±0.019	346	0.32±0.019	104	<0.001
	c	a,b	0.29±0.022	124	0.31±0.022	326	<0.001
C13:0 *iso*	a	None	0.09±0.002	157	0.08±0.002	293	<0.001
	b	None	0.08±0.002	346	0.09±0.002	104	<0.001
	c	None	0.08±0.002	124	0.09±0.002	326	<0.001
	a	b,c	0.09±0.006	157	0.08±0.006	293	<0.001
	b	a,c	0.08±0.006	346	0.09±0.006	104	<0.001
	c	a,b	0.08±0.007	124	0.09±0.007	326	**0.002**
C12:1	a	None	0.10±0.003	157	0.09±0.002	293	<0.001
	b	None	0.09±0.002	346	0.10±0.003	104	<0.001
	c	None	0.09±0.003	124	0.10±0.002	326	*0.074*
	a	b,c	0.10±0.005	157	0.09±0.004	293	<0.001
	b	a,c	0.09±0.005	346	0.10±0.005	104	<0.001
	c	a,b	0.10±0.006	124	0.10±0.006	326	*0.126*
C14:0	a	None	12.36±0.124	157	12.63±0.109	293	**0.002**
	b	None	12.65±0.109	346	12.30±0.127	104	<0.001
	c	None	12.65±0.123	124	12.50±0.113	326	*0.109*
	a	b,c	12.39±0.235	157	12.54±0.228	293	*0.101*
	b	a,c	12.68±0.169	346	12.28±0.178	104	<0.001
	c	a,b	12.61±0.244	124	12.36±0.240	326	**0.015**
C14:1	a	None	1.17±0.026	157	0.93±0.023	293	<0.001
	b	None	0.96±0.026	346	1.11±0.030	104	<0.001
	c	None	0.91±0.029	124	1.05±0.027	326	<0.001
	a	b,c	1.14±0.092	157	0.97±0.091	293	<0.001
	b	a,c	0.98±0.106	346	1.13±0.107	104	<0.001
	c	a,b	1.00±0.116	124	1.11±0.115	326	<0.001
C16:1	a	None	1.27±0.035	157	1.37±0.031	293	<0.001
	b	None	1.33±0.032	346	1.38±0.037	104	*0.057*
	c	None	1.44±0.034	124	1.29±0.031	326	<0.001
	a	b,c	1.31±0.067	157	1.38±0.065	293	**0.004**
	b	a,c	1.33±0.074	346	1.37±0.075	104	0.203
	c	a,b	1.41±0.049	124	1.29±0.046	326	<0.001
C17:0 *iso*	a	None	0.58±0.009	157	0.56±0.008	293	**0.001**
	b	None	0.56±0.008	346	0.58±0.010	104	**0.017**
	c	None	0.56±0.009	124	0.57±0.009	326	*0.158*

**Table 3:** Continued.

FAME	Variant	Other variants in	Mean FAME level ± SE${}^{1}$				$P^{2}$
		the model	($g\;100\;g^{-1}$ milk FA)				
			Absent	n	Present	n	
	a	b,c	0.58±0.011	157	0.56±0.010	293	**0.002**
	b	a,c	0.56±0.013	346	0.58±0.013	104	*0.056*
	c	a,b	0.57±0.015	124	0.57±0.014	326	0.278
C17:1	a	None	0.20±0.004	157	0.20±0.003	293	**0.023**
	b	None	0.20±0.003	346	0.21±0.004	104	**0.024**
	c	None	0.21±0.004	124	0.20±0.003	326	<0.001
	a	b,c	0.20±0.006	157	0.21±0.005	293	0.473
	b	a,c	0.20±0.005	346	0.21±0.005	104	*0.188*
	c	a,b	0.21±0.004	124	0.20±0.003	326	**0.001**
C18:1 *cis*-9	a	None	12.27±0.222	157	13.05±0.196	293	*0.154*
	b	None	12.97±0.196	346	13.48±0.228	104	**0.003**
	c	None	13.15±0.219	124	13.08±0.201	326	0.675
	a	b	13.31±0.315	157	13.17±0.301	293	0.369
	b	a	12.97±0.196	346	13.48±0.228	104	**0.003**
C18:2 *trans-*9, 12	a	None	0.41±0.006	157	0.40±0.005	293	**0.042**
	b	None	0.40±0.005	346	0.40±0.006	104	0.268
	c	None	0.39±0.006	124	0.40±0.005	326	**0.024**
	a	c	0.40±0.007	157	0.40±0.006	293	*0.117*
	c	a	0.39±0.006	124	0.40±0.006	326	**0.046**
C18:2 *cis-*9, *trans*-13	a	None	0.28±0.005	157	0.29±0.005	293	**0.021**
	b	None	0.29±0.005	346	0.29±0.005	104	0.804
	c	None	0.29±0.005	124	0.28±0.005	326	**0.015**
	a	c	0.28±0.006	157	0.29±0.005	293	*0.073*
	c	a	0.29±0.006	124	0.28±0.005	326	**0.045**
C18:2 *cis-*9,* trans-*12	a	None	0.07±0.003	157	0.08±0.003	293	<0.001
	b	None	0.07±0.003	346	0.07±0.003	104	0.768
	c	None	0.08±0.003	124	0.07±0.003	326	<0.001
	a	c	0.07±0.004	157	0.08±0.004	293	**0.001**
	c	a	0.08±0.005	124	0.07±0.005	326	**0.018**
C19:0	a	None	0.14±0.004	157	0.13±0.003	293	**0.036**
	b	None	0.13±0.003	346	0.13±0.004	104	0.217
	c	None	0.13±0.004	124	0.14±0.003	326	**0.026**
	a	c	0.14±0.004	157	0.13±0.004	293	*0.099*
	c	a	0.13±0.004	124	0.14±0.004	326	*0.055*
C20:0	a	None	0.12±0.002	157	0.12±0.002	293	**0.011**
	b	None	0.12±0.002	346	0.12±0.003	104	**0.011**
	c	None	0.12±0.002	124	0.12±0.002	326	0.380
	a	b	0.12±0.003	157	0.12±0.003	293	**0.031**
	b	a	0.12±0.003	346	0.12±0.003	104	**0.029**
C22:5 *cis*-7, 10, 13, 16, 19	a	None	0.12±0.004	157	0.12±0.003	293	**0.041**
	b	None	0.119±0.003	346	0.12±0.004	104	0.387
	c	None	0.121±0.004	124	0.12±0.003	326	0.687

${}^{1}$ Predicted means and standard error of those means derived from GLMM. “Cow age”, “days in milk” and “herd” were fitted to the models as fixed effects. ${}^{2}$ P<0.05 in bold; 0.05<P<0.2 in italics.

**Table 4:** Association between grouped fatty acid levels and *SCD* 3${}^{\prime }$-UTR variation.

FAME	Variant	Other variants in	Mean FAME level ± SE${}^{1}$				$P^{2}$
		the model	($g\;100\;g^{-1}$ milk FA)				
			Absent	n	Present	n	
MCFA	a	None	20.57±0.239	157	21.05±0.211	293	**0.005**
	b	None	21.06±0.213	346	20.54±0.247	104	**0.006**
	c	None	21.11±0.237	124	20.81±0.217	326	*0.093*
	a	b,c	20.62±0.378	157	20.93±0.359	293	*0.090*
	b	a,c	21.11±0.314	346	20.50±0.333	104	**0.003**
	c	a,b	21.04±0.400	124	20.60±0.390	326	**0.026**
Total C18:1	a	None	16.72±0.257	157	16.46±0.226	293	*0.148*
	b	None	16.41±0.227	346	16.86±0.264	104	**0.027**
	c	None	16.55±0.253	124	16.53±0.232	326	0.920
	a	b	16.75±0.309	157	16.55±0.288	293	0.266
	b	a	16.41±0.227	346	16.86±0.264	104	**0.027**
MUFA	a	None	20.41±0.264	157	19.98±0.233	293	**0.018**
	b	None	19.90±0.233	346	20.60±0.271	104	**0.001**
	c	None	20.10±0.261	124	20.09±0.240	326	0.951
	a	b	20.46±0.390	157	20.13±0.374	293	*0.075*
	b	a	19.96±0.269	346	20.61±0.299	104	**0.002**
Total branched FA	a	None	1.633±0.022	157	1.60±0.019	293	**0.018**
	b	None	1.60±0.019	346	1.62±0.022	104	0.300
	c	None	1.59±0.021	124	1.62±0.020	326	0.212
Total UFA	a	None	24.45±0.314	157	24.03±0.277	293	*0.052*
	b	None	23.96±0.277	346	24.64±0.323	104	**0.006**
	c	None	24.15±0.310	124	24.14±0.285	326	0.955
	a	b	24.50±0.416	157	24.17±0.394	293	*0.137*
	b	a	24.00±0.301	346	24.65±0.340	104	**0.010**
Total SFA	a	None	68.46±0.342	157	68.86±0.301	293	*0.093*
	b	None	68.93±0.303	346	68.30±0.352	104	**0.020**
	c	None	68.72±0.337	124	68.77±0.310	326	0.850
	a	b	68.43±0.418	157	68.75±0.391	293	*0.185*
	b	a	68.90±0.316	346	68.29±0.362	104	**0.026**
Total index	a	None	26.32±0.342	157	28.87±0.301	293	*0.058*
	b	None	25.80±0.302	346	26.51±0.352	104	**0.008**
	c	None	26.01±0.338	124	25.99±0.310	326	0.925
	a	b	26.37±0.445	157	26.02±0.420	293	*0.145*
	b	a	25.84±0.326	346	26.52±0.369	104	**0.012**
MUFA index	a	None	23.09±0.315	157	22.60±0.278	293	**0.025**
	b	None	22.52±0.278	346	23.29±0.324	104	**0.002**
	c	None	22.74±0.312	124	22.73±0.286	326	0.965
	a	b	23.14±0.444	157	22.76±0.423	293	*0.086*
	b	a	22.59±0.317	346	23.30±0.354	104	**0.005**
C10:1 index	a	None	9.41±0.184	157	8.01±0.162	293	<0.001
	b	None	8.12±0.177	346	9.10±0.206	104	<0.001
	c	None	7.90±0.198	124	8.66±0.182	326	<0.001
	a	b,c	9.25±0.595	157	8.24±0.589	293	<0.001
	b	a,c	8.27±0.623	346	9.22±0.626	104	<0.001
	c	a,b	8.40±0.711	124	9.07±0.706	326	<0.001

**Table 4:** Continued.

FAME	Variant	Other variants in	Mean FAME level ± SE${}^{1}$				$P^{2}$
		the model	($g\;100\;g^{-1}$ milk FA)				
			Absent	n	Present	n	
C12:1 index	a	None	2.54±0.048	157	2.25±0.042	293	<0.001
	b	None	2.26±0.044	346	2.51±0.051	104	<0.001
	c	None	2.25±0.050	124	2.38±0.046	326	**0.001**
	a	b,c	2.53±0.134	157	2.31±0.132	293	<0.001
	b	a,c	2.30±0.127	346	2.53±0.128	104	<0.001
	c	a,b	2.36±0.164	124	2.47±0.162	326	**0.004**
C14:1 index	a	None	8.69±0.190	157	6.89±0.168	293	<0.001
	b	None	7.05±0.191	346	8.25±0.222	104	<0.001
	c	None	6.73±0.214	124	7.75±0.197	326	<0.001
	a	b,c	8.47±0.748	157	7.17±0.742	293	<0.001
	b	a,c	7.23±0.797	346	8.41±0.800	104	<0.001
	c	a,b	7.37±0.891	124	8.26±0.887	326	<0.001
C16:1 index	a	None	3.23±0.078	157	3.50±0.069	293	<0.001
	b	None	3.39±0.071	346	3.52±0.083	104	**0.047**
	c	None	3.67±0.076	124	3.29±0.069	326	<0.001
	a	b,c	3.34±0.172	157	3.52±0.167	293	**0.002**
	b	a,c	3.40±0.187	346	3.48±0.190	104	0.251
	c	a,b	3.60±0.112	124	3.27±0.106	326	<0.001
C18:1 index	a	None	61.23±0.515	157	61.02±0.454	293	0.542
	b	None	60.75±0.453	346	61.95±0.527	104	**0.003**
	c	None	61.37±0.506	124	60.91±0.464	326	0.234
CLA index	a	None	27.23±0.420	157	27.56±0.370	293	0.259
	b	None	27.25±0.371	346	28.05±0.432	104	**0.016**
	c	None	27.95±0.411	124	27.19±0.377	326	**0.015**
	b	c	27.34±0.446	346	28.00±0.494	104	*0.053*
	c	b	27.97±0.477	124	27.33±0.458	326	**0.049**

${}^{1}$ Predicted means and standard error of those means derived from GLMM. “Cow age”, “days in milk (DIM)” and “herd” were fitted to the models as fixed effects. ${}^{2}$ P<0.05 in bold; 005<P<0.2 in italics.

**Table 5:** Association between milk fatty acid levels and 3${}^{\prime }$-UTR genotypes${}^{1}$.

FAME	Mean FAME level ± SE${}^{2}$ ($g\;100\;g^{-1}$ milk FA)					P
	*aa* (n=68)	*ab* (n=48)	*ac* (n=177)	*bc* (n=48)	*cc* (n=101)	
C10:0	3.36±0.061${}^{a}$	3.20±0.066${}^{\text{ab}}$	3.24±0.051${}^{\text{ab}}$	3.14±0.069${}^{b}$	3.17±0.058${}^{b}$	0.009
C10:1	0.26±0.006${}^{c}$	0.30±0.006${}^{b}$	0.29±0.005${}^{b}$	0.33±0.007${}^{a}$	0.32±0.006${}^{a}$	<0.001
C11:0	0.06±0.003	0.06±0.003	0.06±0.002	0.06±0.003	0.06±0.003	0.047
C13:0* iso*	0.07±0.002${}^{d}$	0.09±0.003${}^{\text{bc}}$	0.08±0.002${}^{c}$	0.10±0.003${}^{a}$	0.09±0.002${}^{\text{ab}}$	<0.001
C12:1	0.09±0.003${}^{c}$	0.10±0.003${}^{\text{ab}}$	0.09±0.002${}^{\text{bc}}$	0.10±0.003${}^{a}$	0.10±0.003${}^{a}$	<0.001
C13:0	0.13±0.004${}^{a}$	0.11±0.005${}^{b}$	0.12±0.004${}^{\text{ab}}$	0.12±0.005${}^{\text{ab}}$	0.12±0.004${}^{b}$	0.018
C14:0	12.93±0.140${}^{a}$	12.35±0.151${}^{b}$	12.59±0.118${}^{\text{ab}}$	12.23±0.158${}^{b}$	12.45±0.134${}^{b}$	<0.001
C14:1* cis*-9	0.80±0.027${}^{c}$	1.01±0.029${}^{b}$	0.97±0.023${}^{b}$	1.20±0.030${}^{a}$	1.14±0.025${}^{a}$	<0.001
C16:1	1.44±0.039${}^{a}$	1.41±0.042${}^{\text{ab}}$	1.33±0.033${}^{b}$	1.30±0.044${}^{\text{bc}}$	1.21±0.037${}^{c}$	<0.001
C17:0* iso*	0.54±0.011${}^{b}$	0.57±0.011${}^{\text{ab}}$	0.56±0.009${}^{\text{ab}}$	0.58±0.012${}^{a}$	0.58±0.010${}^{a}$	0.004
C17:1	0.21±0.004${}^{a}$	0.21±0.004${}^{\text{ab}}$	0.20±0.003${}^{\text{ab}}$	0.20±0.005${}^{\text{ab}}$	0.20±0.004${}^{b}$	0.004
C18:2 *cis-*9,* trans*-13	0.30±0.006${}^{a}$	0.29±0.006${}^{\text{ab}}$	0.29±0.005${}^{\text{ab}}$	0.29±0.007${}^{\text{ab}}$	0.28±0.006${}^{b}$	0.011
C18:2 *cis-*9, *trans-*12	0.08±0.004${}^{a}$	0.08±0.004${}^{a}$	0.07±0.003${}^{a}$	0.07±0.004${}^{\text{ab}}$	0.07±0.004${}^{b}$	<0.001
C20:0	0.13±0.003${}^{a}$	0.12±0.003${}^{\text{ab}}$	0.12±0.002${}^{\text{ab}}$	0.12±0.003${}^{b}$	0.12±0.003${}^{\text{ab}}$	0.044
MCFA	21.55±0.273${}^{a}$	20.66±0.295${}^{b}$	20.96±0.230${}^{\text{ab}}$	20.40±0.309${}^{b}$	20.70±0.260${}^{b}$	0.002
MUFA	19.87±0.300${}^{b}$	20.24±0.323${}^{\text{ab}}$	19.92±0.252${}^{b}$	20.88±0.339${}^{a}$	20.06±0.285${}^{\text{ab}}$	0.020
MUFA index	22.50±0.359${}^{\text{ab}}$	22.87±0.387${}^{\text{ab}}$	22.55±0.302${}^{b}$	23.64±0.405${}^{a}$	22.70±0.341${}^{\text{ab}}$	0.036
C10:1 index	7.17±0.196${}^{c}$	8.56±0.211${}^{b}$	8.20±0.165${}^{b}$	9.67±0.221${}^{a}$	9.19±0.186${}^{a}$	<0.001
C12:1 index	2.08±0.051${}^{d}$	2.40±0.055${}^{\text{bc}}$	2.280±0.043${}^{c}$	2.61±0.057${}^{a}$	2.47±0.048${}^{\text{ab}}$	<0.001
C14:1 index	5.80±0.192${}^{c}$	7.54±0.207${}^{b}$	7.160±0.161${}^{b}$	8.99±0.216${}^{a}$	8.43±0.182${}^{a}$	<0.001
C16:1 index	3.71±0.085${}^{a}$	3.57±0.092${}^{\text{ab}}$	3.387±0.072${}^{b}$	3.33±0.096${}^{\text{bc}}$	3.09±0.081${}^{c}$	<0.001
CLA index	27.79±0.470	27.82±0.507	27.376±0.395	27.80±0.530	26.63±0.447	0.037

${}^{1}$ The genotypes with a frequency greater than 5 % were analysed. Genotype *bb* (n=8) occurred at a frequency of 1.78 % and hence was not analysed. ${}^{2}$ Predicted means and standard error of those means derived from GLMM. “Cow age”, “days in milk” and “herd” were fitted to the models as fixed effects. Means within a row that do not share a superscript letter are separated by Bonferroni test at P<0.05.

Table 5 summarizes the associations revealed between the composition of milk fat and the *SCD* genotypes from the 3${}^{\prime }$-UTR variants. Results are only presented if an association was found. The frequency of the *bb* genotype was low (1.8 %). Thus *bb* cows were not included in the analyses. The effects of c.*2066T/C/G and c.*1783A/G on milk fat composition were observed as the difference between *bc* and *cc* genotype cows, with there being less C16:1, C17:1, C18:2 *cis*-9, *trans*-13 and C18:2 *cis*-9, *trans*-12 in the milk from *cc* cows.

## 4 Discussion

The enzyme SCD is a rate-limiting enzyme in the biosynthesis of many monounsaturated FAs (Ntambi et al., 2004), and sequence variation in *SCD* has been revealed to affect milk fat composition. The *SCD* gene is therefore considered to be a useful candidate gene for use in breeding programmes targeted at improving the nutritional value of milk. The most commonly described variation in *SCD* is the variant c.878C/T located in exon 5. It underpins the substitution of the amino acid valine (V) with alanine (A) at amino acid position 293 in the SCD protein. The function of SCD is likely to be affected by p.A293V because it is located in the third histidine-rich region of the enzyme. This histidine-rich region has been revealed to be important for the catalytic activity of the enzyme (Shanklin et al., 1994).

There was linkage between c.878C/T and other variants. Taniguchi et al. (2004) identified linkage between three nucleotide substitutions c.702A/G, c.762T/C and c.878C/T in exon 5 of Japanese black cattle. Based on these three variants, they described the haplotype p.293V (GCT) and p.293A (ATC). This linkage was also found by Baeza et al. (2013) with the variant g.10153A> G (equivalent to c.702A/G here) being in complete linkage disequilibrium with c.878C/T in the beef cattle they studied. In this study, three nucleotide variations in exon 5 (c.702A/G, c.762T/C and c.878C/T), one variant in intron 5 (c.880+105A/G) and four variants in the 3${}^{\prime }$-UTR (c.*1883C/T, c.*1984G/A, c.*1783A/G and c.*2066T/C/G) were revealed. There was linkage between c.702A/G, c.762T/C, c.878C/T, c.880+105A/G, c.*1883C/T and c.*1984G/A.

In an in vitro study, Enoch et al. (1976) revealed that the acyl-CoA derivatives with 12 to 19 carbon atoms were required as substrates for SCD enzyme activity. Schennink et al. (2008), Kgwatalala et al. (2009b) and this research (Table 1) all suggested that *SCD* p.A293V has effects on individual FA and FA index levels, and especially in this study on the level of C10:0, C10:1, C12:1, C14:0, C14:1 and C16:1, as well as the C10:1 index, C12:1 index, C14:1 and C16:1 index levels. In addition, Enoch et al. (1976) found that the SCD enzyme has substrate specificity, with a preference for longer-chain FAs. However, the effect of p.A293V on C18 FA levels (such as the C18:1 *trans*-11, C18:1 *cis*-9, C18 index) was not confirmed here.

The effect of p.A293V on C18 FA levels could be influenced by different factors, including seasonal variation, the impact of other genes, variation between breeds and the stage of lactation. Duchemin et al. (2013) revealed that the p.293V allele was negatively associated with C18:1 *trans*-11 and that this negative effect was larger in summer than in winter. Schennink et al. (2008) investigated the joint effect of *SCD* and *DGAT1* variation and suggested that the genetic variation explained by *DGAT1* p.232K and by *SCD* p.293A is additive with respect to their effect on C16, C18 and CLA levels. Moioli et al. (2007) revealed that p.A293V affected C10:1, C14:1, C16:1, C10 index and C14 index levels in Piedmontese (n=81), Jersey (n=75) and Valdostana (n=730) cows, but the effect of p.293A on C18 levels was only observed when the variant frequency was high (i.e. when at a frequency of 0.94 in Jersey cattle and 0.65 in Valdostana cattle). With a lower frequency of occurrence of p.293A (i.e. 0.42 in Piedmontese cattle), the effect of *SCD* variation on C18 levels was not significant. Mele et al. (2007) investigated the effect of p.293A and lactation stage on C18:1 *cis*-9 FA levels. A negative effect was observed when the DIM increased (P<0.01), and that p.293A occurrence (the frequency was 0.57) had a positive effect on the C18:1 *cis*-9 FA level (P<0.05). Finally Carvajal et al. (2016) revealed associations between *SCD* p.A293V genotypes and C6:0, C8:0, C10:0, C11:0, C14:0, C16:0, C18:0, C20:0, C16:1, C18:1 *cis*-9, linoleic acid, α-linolenic acid and CLA *cis*-9 *trans*-11 levels.

In our study, a higher frequency of the p.293A allele (59.9 %) was observed in the Kiwicross™ cows. This is consistent with the findings of Schennink et al. (2008), Kgwatalala et al. (2009b) and Mele et al. (2007), who reported similar results, with frequencies of 73 % in Dutch HF cows, 69 % in Canadian HF cows and 57 % in Italian HF cows, respectively.

The 3${}^{\prime }$-UTR can play a critical role in translation termination and post-transcriptional gene expression (Barrett et al., 2012). Baeza et al. (2013) reported that the 3${}^{\prime }$-UTR variant g.15001A>G (equivalent to c.*1783A/G here) affected meat fat composition (C14:1 level, P<0.05) in their Argentinian Brangus beef cattle. In the results reported here, after differentiating the p.293A variant to b and c (based on 3${}^{\prime }$-UTR typing), the different effects of the variants on long-chain FA levels were revealed. The decline in C16:1, C17:1, C18:2 *cis*-9, *trans*-13 and C18:2 *cis*-9, *trans*-12 FA levels and C16:1 index level was associated with the presence of variant c. The presence of variant b was associated with an increase in C18:1 *cis*-9 and total C18:1 FA levels, MUFA levels, C18:1 index level and MUFA index level. At the level of genotype (Table 5), there was a significant difference between *aa* and *cc* cows in C16:1, C17:1, C18:2 *cis*-9, *trans*-13 and C18:2 *cis*-9, *trans*-12 FA levels. In addition, there was a significant difference between *aa* and *bb* cows for C20:0 levels and MUFA levels. This suggests the 3${}^{\prime }$-UTR (specifically the substitutions c.*1783A/G and c.*2066T/C/G) is better able to resolve the effect of *SCD* on milk FA levels. In this respect, Kgwatalala et al. (2009b) regarded the effect of different lactation stages on milk MUFA was more important than p.A293V, and this along with the results presented here suggests more research into *SCD* variation and lactation stage is needed to gain clarity into what is driving variation in FA levels.

The influence of 3${}^{\prime }$-UTR variation was also described by Kgwatalala et al. (2009a) in 46 Holstein and 35 Jersey cows in Canada. In their study, three haplotypes H1, H2 and H3 (equivalent to the variants c, b and a here) were identified with the frequency 67.1 %, 2.3 % and 30.6 % respectively. Significant differences were only found in two milk FA levels, with their H1H1 cows producing more C10:1 and C12:1 than the H3H3 cows (similar results were observed when comparing *cc* and *aa* cows here). Moreover, Kgwatalala et al. (2009a) reported that an internal ribosome entry site (IRES) could be found in the c variant only. The presence of an IRES motif may ultimately affect SCD protein turnover or the quantity of the SCD enzyme produced, because it could enhance translation of the constituent mRNA. Kgwatalala et al. (2009a) suggested that the nucleotide variation in 3${}^{\prime }$-UTR region might lead to the absence of the IRES in the a and b variants.

With a low frequency of b in their Holstein (0.054) and Jersey (0.000) cattle, Kgwatalala et al. (2009a) suggested that milk fat composition was not affected by variant b significantly. Therefore, the variation in C10:1 and C12:1 FA levels in their study could be due to either the variation in the 3${}^{\prime }$-UTR (c.*1783A/G) or the variation in exon 5 (c.878C/T). If this was true, then the effect of c on milk fat composition should be the opposite of the effects of a, b or a+b. In the Kiwicross™ cows studied here the frequency of b was 0.124, and opposite effects were observed between variant c and the other two variants for C16:1, C18:1 *cis-*9, C18:2 *trans-*9, 12, C18:2 *cis*-9, *trans*-12, C18:2 *cis*-9, *trans*-13, C19:0 FA levels and C16:1 index levels (Tables 3 and 4). At the genotype level (Table 5), the *cc* cows produced more C10:1 and C12:1 FA than the *aa* cows. This is similar to what was reported by Kgwatalala et al. (2009a). Moreover, the *ab* cows produced less C10:1 and C14:1 *cis*-9 FA but more C18:2 *cis*-9, *trans*-12 and C16:1 FA than the *cc* cows.

The effect of *SCD* on gross milk traits (milk yield, fat and protein percentage) is still disputed. Macciotta et al. (2008) investigated 313 Italian Holstein cows and found that their *VV* cows (referring to p.A293V) had higher milk yields and protein yields than their *VA* and *AA* cows. The associations they found appear to be consistent across different stages of lactation. However, Mao et al. (2012) and Signorelli et al. (2009) reported a significant negative effect of the V allele on milk yield in Chinese Holstein, Piedmontese and Valdostana breeds. In addition, Schennink et al. (2008) did not find any significant associations between p.A293V and milk traits in Dutch Holstein, a result that was consistent with our findings here in and Kiwicross™ cows.

## Data Availability

The original data are available upon request to the corresponding author.
